# A simulation-based empirical study on the role of aviation logistics in driving high-quality and sustainable regional economic development: Focusing on dynamic mechanisms and key factors

**DOI:** 10.1371/journal.pone.0323110

**Published:** 2025-05-08

**Authors:** Lijun Liang, Peirong Chen

**Affiliations:** 1 School of Business, Beijing Information Science and Technology University (BISTU), Beijing, China; 2 School of Management Science and Engineering, Beijing Information Science and Technology University (BISTU), Beijing, China; Air Force Engineering University, CHINA

## Abstract

This study incorporates aviation logistics equipment into the analysis, considering current policy trends and development hotspots. By exploring the intricate relationship between aviation logistics and the regional economy, a System Dynamics model is created to identify the dynamic mechanisms and key factors that drive high-quality and sustainable regional economic development through aviation logistics. The primary objective is to establish a framework for resource allocation and policy adjustments tailored to regional needs. To validate the proposed framework, an empirical analysis of Sichuan Province is conducted. The findings indicate that: (1) Aviation logistics significantly contributes to regional economic development; (2) Aviation logistics equipment, a crucial component of the aviation logistics supply subsystem, demonstrates notable stability compared to demand and plays a pivotal role in fostering regional economic development; (3) Aviation logistics equipment software, in contrast to hardware, has a more pronounced impact on regional economic development and structural optimization. This study offers both theoretical and practical contributions: Firstly, it integrates aviation logistics equipment into the complex system framework, addressing a gap in the literature by defining and categorizing its role within the framework. Secondly, the dynamic model developed provides theoretical support for regional management and policy-making, facilitating improved resource allocation and policy optimization for high-quality and sustainable regional economic development. Finally, the study presents practical policy recommendations, making it highly valuable in practice based on the Sichuan case.

## Introduction

Economic globalization has increasingly positioned aviation transport as a crucial driver of regional competitiveness, with aviation logistics emerging as a strategic catalyst for economic development [1]. This is particularly evident in the post-pandemic recovery phase and the rapid growth of emerging sectors such as cross-border e-commerce [2]. In China, the aviation logistics market has expanded rapidly, with aviation freight volumes consistently increasing [3]. According to data from the Civil Aviation Administration of China (CAAC), aviation freight and mail volumes in China reached 7.354 million tons in 2023, reflecting a 21% increase compared to the previous year. This growth has significantly contributed to the national economic recovery and the stability of the supply chain.

However, despite the robust momentum of China’s aviation logistics development, several challenges persist [4]. For instance, China’s aviation hubs demonstrate limited competitive strength, with only three international airports ranked among the top 50 in the OAG 2023 Super Hub Airport Index, compared to 11 airports in the United States. Additionally, the aviation logistics network in China remains underdeveloped, as evidenced by the 4,334 domestic routes in 2023, while the international route network comprises only 336 routes. These limitations are likely to impede the further development of the regional economy.

These challenges primarily arise from insufficient supply capacity, which is due to limitations in aviation logistics infrastructure, the scale of route networks, and other factors. In response, China has increasingly prioritized the development of logistics equipment, now recognized as a critical pillar for advancing the logistics sector. The government has introduced a series of policies aimed at enhancing logistics equipment. For instance, the “Action Plan for Large-Scale Equipment Renewal in Transportation,” issued in 2024 by the Ministry of Transport and thirteen other departments, calls for the upgrading and modernization of multimodal transport stations and intermodal facilities. It also emphasizes the need to improve other logistics infrastructure at key national logistics hubs, major cold chain logistics bases, national demonstration logistics parks, and suburban warehouse hubs. These initiatives are designed to stimulate regional economic development by strengthening logistics equipment. The development of aviation logistics equipment plays a crucial role in enhancing the efficiency and capacity of aviation logistics system, significantly supporting global trade flows and stimulating regional economic development [5]. Therefore, strengthening aviation logistics equipment is not merely a sector necessity but a strategic element for encouraging sustainable regional economic development.

Aviation logistics is a complex sector made up of multiple components. To fully harness the potential of aviation logistics in fostering regional economic development, several key questions must be addressed: How does aviation logistics contribute to regional economic development? What factors influence this contribution? How can limited resources be allocated to areas that maximize the impact of aviation logistics on the regional economy? How should the development of aviation logistics and the regional economy (AL&RE) be assessed? When considering aviation logistics equipment, what are its specific definitions and scope, and what role does it play in promoting regional economic development?

To address these issues, this study investigates the driving mechanisms and key factors of aviation logistics in promoting regional economic development. The novel contributions of this study are as follows: (1) This study integrates aviation logistics equipment into the analytical framework and constructs an AL&RE complex system. In doing so, it further clarifies the connotation of aviation logistics equipment and categorizes it. This work enhances the understanding of the complex interactions between AL&RE and fills a gap in the literature regarding the role and position of aviation logistics equipment within this mechanism, providing a theoretical contribution to the field. (2) Given that the System Dynamics (SD) method is effective in modeling nonlinear dynamics, system feedback, behavioral responses, and alternative scenarios [6], this study develops an SD model to simulate and analyze the mechanism through which aviation logistics drives regional economic development. This approach, from a dynamic perspective, offers valuable insights for the study of complex systems. (3) To validate the applicability of the model, this study conducts an empirical simulation using Sichuan Province as a case study, performing result analysis and sensitivity analysis. This reveals the key driving factors of aviation logistics in regional economic development. The analysis not only helps assess the dynamic relationship and policy effects between AL&RE under current policies in Sichuan but also provides empirical evidence for policy adjustment and formulation, offering significant practical implications. (4) This study presents a dynamic model that demonstrates how aviation logistics can stimulate regional economic development. This model serves as a guide for formulating policies designed to promote high-quality, sustainable regional economic development through aviation logistics across various regions. It provides valuable insights for future research and policy simulations and adjustments in other areas.

In the remaining section of this part, a literature review is conducted, followed by the construction and analysis of the AL&RE complex system, along with the connotation and classification of aviation logistics equipment. Part 2 outlines the research materials and methods, including the definition of system boundaries, formulation of system hypotheses, construction of a causal feedback model and system flow diagram, selection of the empirical region, data collection, and variable setting. Part 3 presents the research results and discussion, encompassing model validation, simulation results analysis, sensitivity analysis, and policy recommendations. Part 4 concludes with the findings and directions for future research.

## Literature review

### Interaction between AL&RE

The economic impacts of aviation logistics manifest both directly, through activities within the aviation sector, and indirectly, through increased spending and broader economic benefits associated with improved access to resources, markets, technology, and economies of scale. Consequently, economic activity drives and stimulates aviation logistics [7]. Given this close relationship, the interaction between AL&RE, along with the key interrelated factors, has attracted significant attention. These factors include the driving force of aviation logistics on the regional economy and the stimulating effect of regional economic development on the growth of aviation logistics.

The role of aviation logistics in driving regional economic development has been extensively examined. For instance, Sheard [8] emphasized that airport scale significantly positively impacts the number of local enterprises, population size, employment rate, and GDP growth. Chen et al. [9] quantified the spatial spillover effect of airports on the regional economy, confirming that an increase in aviation cargo volume and flight frequency significantly promotes GDP growth. Zhou et al. [10] through regression analysis of panel data from 91 prefecture-level cities in China, found that the development of aviation logistics infrastructure has a significant positive effect on economic growth in inland regions and secondary gateway cities. Njoya and Ragab [11] utilized a general equilibrium framework to investigate the economic impact of increased investment in Egypt’s aviation logistics infrastructure. Their results also demonstrated that the effect of expanding aviation logistics infrastructure is both significant and robust. He et al. [12] employed complex network theory alongside an econometric framework, identifying the crucial role of China’s domestic aviation cargo network in local economic development.

The impact of regional economy on the advancement of aviation logistics has also been widely examined in the literature. For example, Li et al. [13] utilized network theory to illustrate that China’s aviation logistics network is profoundly influenced by GDP and industrial structure. Hakim and Merkert [14] employed a fixed effects model combined with a three-step error correction mechanism (ECM) to explore the factors driving aviation logistics development in South Asia. Their results emphasized per capita income, investment, and industrial structure as crucial contributors to the growth of aviation logistics.

These studies highlight the interactions between AL&RE, emphasizing that aviation logistics not only drives regional economic development but that the regional economy also significantly influences the growth of aviation logistics. These impacts arise from the interplay of various factors. However, there is a lack of systematic consideration and integration of these variables. Most studies concentrate solely on validating the effects of individual variables, making it difficult to identify the key factors that most significantly influence system evolution.

### AL&RE from a complex systems perspective

As the understanding of the interaction between AL&RE deepens, scholars have transitioned from a static viewpoint to examining the internal complexities and dynamism within both AL&RE systems.

Torres et al. [15] stated that complex systems consist of fundamental units and their interactions. Zhang and Graham [7] highlighted that complex dynamics exist within both AL&RE systems. Consequently, AL&RE have been acknowledged as two complex systems. Guzzo et al. [16] suggested that SD assists in managing heightened complexity by promoting closed-loop thinking, identifying the causal structures that underlie behavior, and enabling proactive experimentation with systems through simulation. Therefore, SD is regarded as an effective tool for identifying and analyzing system complexities. In research on AL&RE from a complex systems perspective, SD has been widely applied.

In the aviation logistics system, Rocha [17] emphasized that this system is highly dynamic over temporal scales ranging from minutes to years. This dynamic behavior not only characterizes the system’s evolution but also influences its functioning. He and Wang [18] employed SD method and system simulation techniques to analyze changes in airport transport corridor systems under three economic growth scenarios: sustained rapid growth, economic slowdown, and economic cycle fluctuations. Peng et al. [19] constructed an SD model for airport environmental carrying capacity (AECC), simulating the model and establishing different scenarios to evaluate suitable airport development modes, considering sustainable development needs. Zhao and Wu [2] developed an SD model of the multi-airport logistics system in the Jing-Jin-Ji region, using simulations of three different COVID-19 impact scenarios to examine the system’s sustainable development.

Within the regional economic system, Anderson [20] asserted that the economy, as evolving complex systems, exhibit dynamic complexity, primarily characterized by the intricate interactions and evolution of internal factors. Gao et al. [21] combined SD and material flow analysis (MFA) to establish a framework for the comprehensive evaluation of the regional economy and further developed nine development scenarios. Jiang et al. [22] employed SD models to design four future policy scenarios, simulating changes in the regional economic development of provinces along the Yellow River Basin.

These studies emphasize that many researchers have already regarded both AL&RE as complex systems, considering their internal dynamics and complexities. However, current research tends to concentrate on individual systems, with limited focus on the complex interactions between AL&RE from a complex systems perspective.

In conclusion, as two complex systems, AL&RE interact through various interrelated variables, demonstrating a complex interplay. Nevertheless, research on this intricate interaction remains confined to static, isolated levels. The study of AL&RE has primarily been approached from a separate, isolated viewpoint, and the dynamic interaction mechanisms between the two systems have not been sufficiently explored. Furthermore, research on the role of aviation logistics in fostering regional economic development has largely been restricted to identifying influencing variables, lacking a comprehensive understanding of the driving mechanisms and pathways. Additionally, aviation logistics equipment, a vital driver of the new aviation logistics system strongly supported by national policies, has been largely overlooked in existing studies.

This study addresses these gaps by constructing a complex system that captures the intricate interactions between AL&RE. It incorporates aviation logistics equipment to analyze its role and interactions with other internal factors and subsystems. Based on this complex system framework, an SD model is developed to explore how aviation logistics drives regional economic development, providing a comprehensive understanding of the underlying mechanisms and key driving factors. Finally, the feasibility of this framework is empirically validated through a case study of Sichuan Province. The simulation model analyzes and forecasts the development of AL&RE, as well as the effectiveness of current policies. Additionally, sensitivity analysis is conducted to identify the key factors driving the regional economy through aviation logistics, and policy recommendations are proposed based on the findings.

### Analysis of the AL&RE complex system and the connotation of aviation logistics equipment

Before analyzing the driving mechanisms and key factors through which aviation logistics impact the regional economy, it is essential to first identify the complex system formed by AL&RE, along with the intricate interactions and nonlinear relationships among its constituent components. Furthermore, regarding aviation logistics equipment, it is essential to define its position, role, and interrelationships with other factors within the AL&RE complex system. Only by clarifying these aspects can the research be effectively conducted.

### Analysis of AL&RE complex system

Many scholars have recognized logistics, including industry-specific logistics, and the regional economy as interconnected nonlinear complex systems from a systems perspective [23,24]. Aviation logistics can also be viewed as a nonlinear complex system interacting with the regional economy due to its dynamic connections across transportation networks, supply chains, infrastructure, and economic impacts on trade, employment, and competitiveness [25].

The aviation logistics system can be specifically divided into two subsystems: demand and supply. These subsystems exhibit distinct functions and roles while remaining interdependent and closely interacting [26]. The aviation logistics demand subsystem primarily reflects factors such as fluctuations in demand, which represent the regional need for aviation logistics services. The aviation logistics supply subsystem includes elements like transportation vehicles, storage facilities, infrastructure development, and technological support, all of which collectively determine the capacity, quality, and efficiency of logistics services. Optimizing the supply subsystem directly influences the demand subsystem’s ability to meet market requirements.

The regional economic subsystem, signifies the level of economic development within the region, incorporating essential factors such as economic output and industrial structure [27]. These three subsystems interact and support one another, creating a complex dynamic system. Changes in the regional economic subsystem are affected not only by the aviation logistics supply but also by the modifications in the demand subsystem.

In addition, the internal environment of the AL&RE complex system is influenced by changes in the external environment, including policies, finance, infrastructure, markets, and other external factors [28]. These external elements interact with the internal components of the system, such as information and capital flows, which, to some extent, affect the evolution of the complex system.

As an emerging field, the concept of aviation logistics equipment lacks a clear and universally accepted definition, with limited comprehensive classifications. This study argues that the definition should extend beyond physical components to encompass its broader role within the interactive context of AL&RE. Since aviation logistics equipment interacts with various other aviation logistics and economic factors, focusing solely on the equipment may overlook its wider dynamic impact. Therefore, a comprehensive definition must account for its role within the AL&RE complex system.

**Fig 1:**
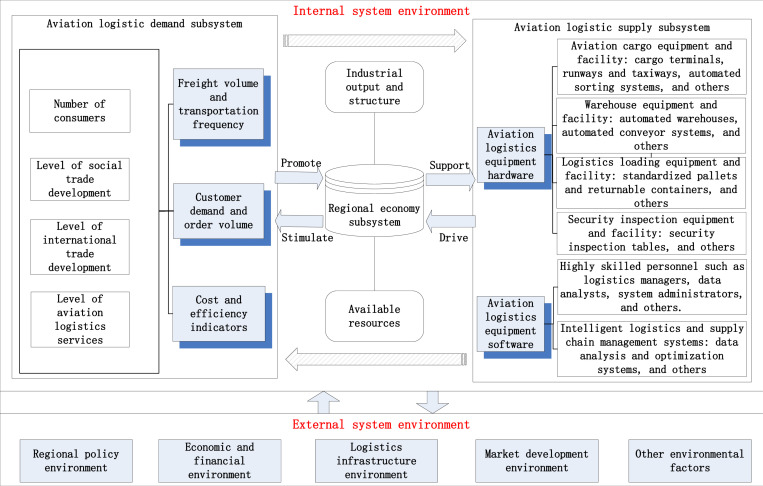
Model of the AL&RE complex system and the interaction mechanism among its internal components.

Given that equipment is generally classified within the supply domain [29,30], this study categorizes it within the aviation logistics supply subsystem. Based on this classification, a complex interaction mechanism model between AL&RE is developed, as shown in Fig 1.

As illustrated in Fig 1, a strong interrelationship and interaction exist among the aviation logistics demand subsystem, the aviation logistics supply subsystem, and the regional economic subsystem. The growth in aviation logistics demand stimulates regional economic output and structural optimization, thereby invigorating regional economic vitality. This results in an increase in resources available for investment in the development of aviation logistics. These resources are primarily allocated toward infrastructure construction, equipment upgrades, and talent development, thus driving the enhancement of aviation logistics supply capacity. Simultaneously, improvements in aviation logistics supply capacity provide substantial and efficient transportation support for further regional economic development. The development of the regional economy, in turn, stimulates external trade activities, increases consumer purchasing power, and attracts resident populations. These activities undoubtedly foster further increases in aviation logistics demand.

Consequently, these three subsystems form a dynamic system characterized by mutual drivers and co-evolution. Moreover, the external environment generates certain disturbances within the complex system through information transmission, material exchange, and other factors.

### The connotation of aviation logistics equipment

Currently, the definitions and classifications of aviation logistics equipment are not clearly established. To ensure that the definitions and classifications proposed in this study are reasonable, this research comprehensively examines the role and position of aviation logistics equipment within the AL&RE complex system, while referencing key aviation logistics equipment and facilities currently being explored in the academic field [31,32]. Additionally, the study investigates aviation logistics equipment mentioned in policy documents such as China’s ‘Action Plan for Large-Scale Equipment Renewal in Transport’ and the ‘14th Five-Year Plan for Aviation Logistics’. After synthesizing these efforts and drawing an analogy with classification methods used for other complex equipment [33,34], this study categorizes aviation logistics equipment into two main types: hardware and software. The specific classification and internal factors are detailed in Table 1.

**Table 1:** The classification of aviation logistics equipment.

Categorization	Factor	Component
Hardware	Aircraft and transportation equipment and facility	Aircraft
		Refrigerated vehicles and cold chain transport tools
		Border port facilities and rail interchange equipment
	Intelligent warehousing equipment and facility	Automated sorting systems
		Stacking machines and electric forklifts
		Smart vertical warehouses
	Logistics loading equipment and facility	Standardized pallets and returnable containers
	Security inspection equipment and facility	X-ray machines
		Security inspection tables
Software	Intelligent scheduling and management systems	Flight scheduling systems
		Intermodal transport scheduling systems
	Intelligent warehousing and logistics management systems	warehouse management platforms
		Intelligent warehouse management systems
	Data analysis and optimization systems	Big data analytics platforms
		Artificial intelligence optimization systems
	Cold chain monitoring systems	Temperature and humidity monitoring systems
		Cold chain tracking and alert systems
	Equipment and facility intelligent control systems	Automated equipment control systems
		Intelligent material tracking systems
	Logistics talents	Technical development talent
		Operational talent
		Support talent
		Product talent
		Specialized talent

The definition of aviation logistics equipment in this study is as follows: it refers to the essential facilities and resources that enhance service levels in aviation logistics and optimize operational efficiency. At its core, aviation logistics equipment serves as a fundamental enabler, addressing the market’s demand for efficient aviation logistics services.

Specifically, aviation logistics equipment hardware includes all physical devices, mechanical equipment, transportation tools, and infrastructure that support aviation logistics operations. These hardware components must exhibit characteristics such as intelligence, sustainability, and automation to improve overall logistics efficiency and reduce energy consumption [35]. By providing essential foundational support, the equipment hardware ensures the efficient and accurate operation of the aviation logistics system, serving as the physical infrastructure and operational backbone of aviation logistics operations.

Aviation logistics equipment software encompasses all digital and intelligent systems and platforms that optimize, control, schedule, and manage both logistics hardware and the cargo transportation process. It also includes the development of software and the specialized personnel required to support its operation. These professionals have distinct roles, each contributing to various functions within the aviation logistics sector [36].

The hardware and software components are mutually reinforcing and interdependent. Hardware provides the physical platform and technical infrastructure for software operations, while software enhances management and operational processes, ensuring the effective performance of hardware. Consequently, the coordination and integration of these components are crucial in boosting the supply capacity of aviation logistics, directly impacting both the development level of aviation logistics and regional economic competitiveness.

## Materials and methods

**Fig 2:**
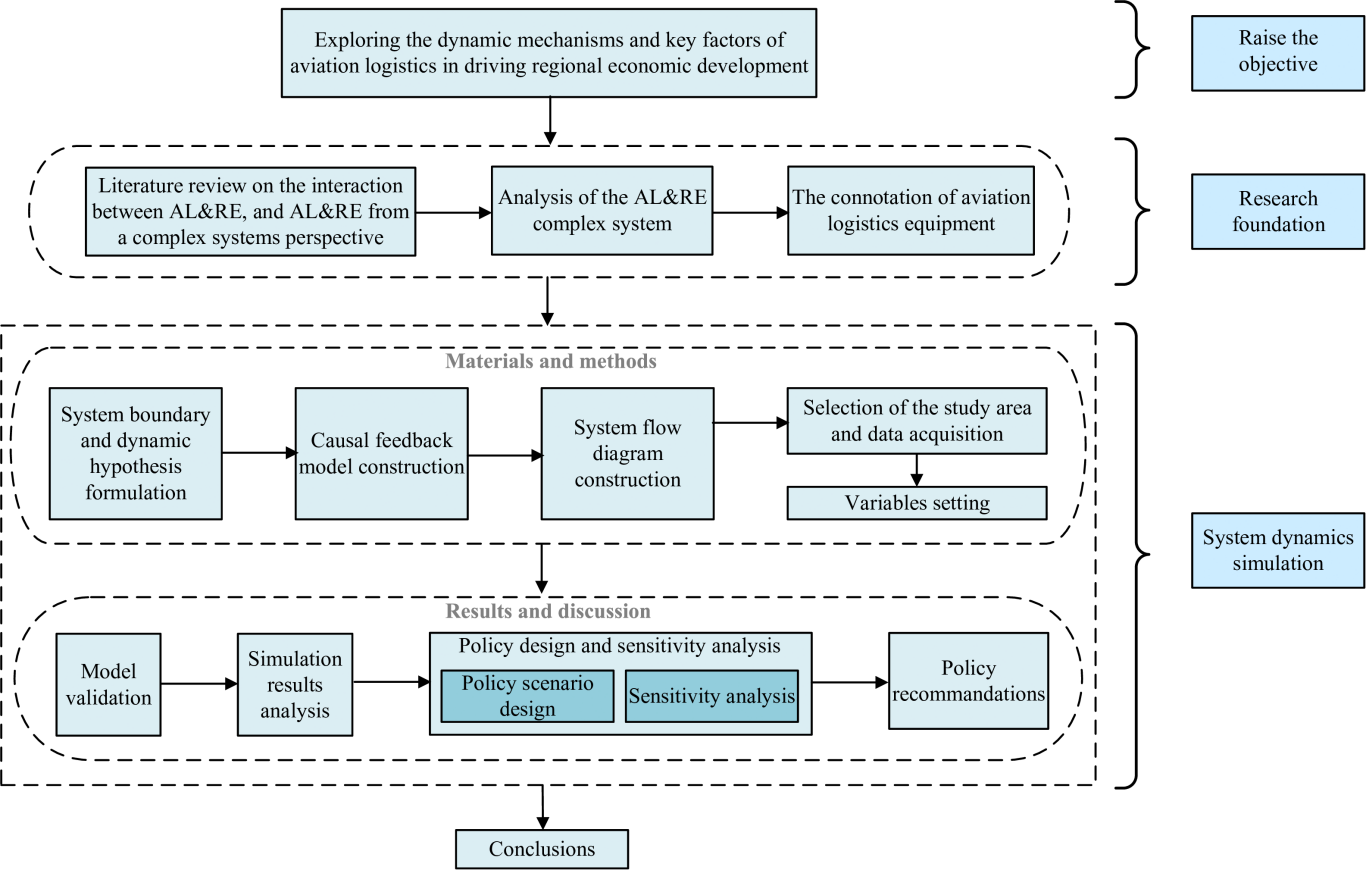
Research framework developed for this study.

Given the AL&RE complex system, traditional linear models are insufficient for effectively analyzing the processes and mechanisms through which aviation logistics contribute to regional economic development. SD, which integrates feedback control theory and computer simulation, is well-suited for uncovering the dynamic structures and underlying mechanisms of these systems [37]. Consequently, this study opts to employ the SD method to develop a dynamic model that illustrates the mechanisms and key factors through which aviation logistics enhance regional economic development. To improve the clarity of this study, a comprehensive research framework has been developed, as shown in Fig 2.

### System boundary and dynamic hypothesis

Clear system boundaries are crucial for model construction [38]. Based on the developed AL&RE complex system model and its internal components and subdivisions, this study identifies three subsystems: aviation logistics demand, aviation logistics supply, and regional economy. The process through which aviation logistics propels regional economic development can be viewed as an evolutionary process of the AL&RE complex system. In this context, the demand and supply subsystems function as input systems, while the regional economic subsystem acts as both the output system and performance metric. The exchange of information and materials among these subsystems enhances the impact of aviation logistics on regional economic development, while feedback from the regional economy, in turn, further accelerates the advancement of aviation logistics. This transfer of information from the input systems to the output system, along with the feedback from the output system to the input systems, is crucial in driving the transformation and progression of the overall system. The dynamic hypothesis offers a framework for clarifying the problematic behavior of a system [39]. To improve the clarity of the study, the following hypotheses are proposed:

Hypothesis 1 (H1): To ensure the operational feasibility of the study, the measurement of aviation logistics supply capacity in this research concentrates exclusively on aviation logistics equipment. Specifically, the scope of aviation logistics equipment is defined according to the definitions and classifications established in this study.

Hypothesis 2 (H2): To streamline the model and maintain focus on the core objectives of this study, external factors and environmental influences on the AL&RE complex system are excluded from the model construction.

### Causal feedback model construction

**Fig 3:**
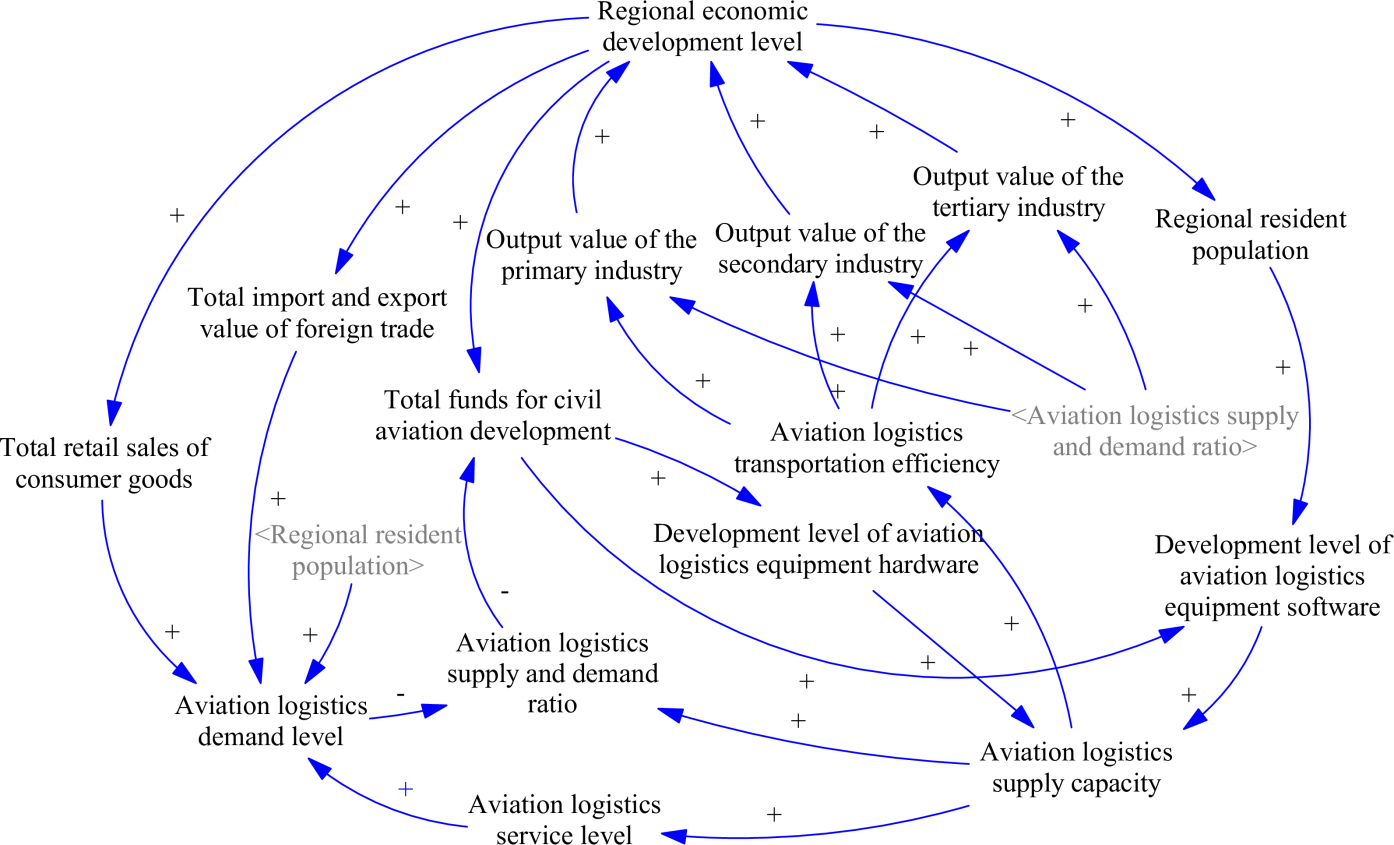
The causal feedback model.

System behavior changes are determined by the composition and interactions of its internal elements, which can be represented using a causal feedback model [40]. Building on the established AL&RE complex system framework and defined system boundaries, this study develops a causal feedback model consisting of 29 causal chains, as shown in Fig 3.

Numerous factors within the system interact to propel regional economic development, creating seven feedback loops. This dynamic is mainly driven by the interactions and feedback between regional economic development and the supply and demand of aviation logistics, along with the interconnections within the aviation logistics sector. These feedback loops can be categorized as follows:

Loop 1: Regional economic development level→+Regional resident population→+Development level of aviation logistics equipment software→+Aviation logistics supply capacity→+Aviation logistics transportation efficiency→+Output value of the primary, secondary, and tertiary industries→+Regional economic development level
Loop 2: Regional economic development level→+Total funds for civil aviation development→+Development level of aviation logistics equipment hardware and software→+Aviation logistics supply capacity→+Aviation logistics transportation efficiency→+Output value of the primary, secondary, and tertiary industries→+Regional economic development level

Feedback loops 1 and 2 primarily illustrate the interactions and driving processes between the regional economic subsystem and the aviation logistics supply subsystem. Loop 1 indicates that advancements in regional economic development create more employment opportunities and elevate living standards, thereby attracting additional migrants and driving population growth [41]. This influx enhances the labor force and technical expertise available to the aviation logistics industry. As a component of aviation logistics equipment software, it boosts the sector’s supply capacity. As supply capacity increases, transportation efficiency improves, leading to higher output across all sectors and providing robust support for sustained regional economic development.

Loop 2 shows that as regional economic development progresses, local government fiscal support increases, enhancing investments in civil aviation development funds. These funds are utilized not only to upgrade and expand aviation logistics equipment hardware but also to support software development, thereby enhancing logistics supply capacity. With improved logistics supply capacity, transportation efficiency is optimized, fostering industrial growth and accelerating regional economic development.

Loop 3: Regional economic development level→+Total import and export value of foreign trade→+Aviation logistics demand level → -Aviation logistics supply and demand ratio→+Output value of the primary, secondary, and tertiary industries→+Regional economic development level
Loop 4: Regional economic development level→+Total retail sales of consumer goods→+Aviation logistics demand level → -Aviation logistics supply and demand ratio→+Output value of the primary, secondary, and tertiary industries→+Regional economic development level
Loop 5: Regional economic development level→+Regional resident population→+Aviation logistics demand level → -Aviation logistics supply and demand ratio→+Output value of the primary, secondary, and tertiary industries→+Regional economic development level

Feedback loops 3, 4, and 5 illustrate the interactions and influencing processes between the regional economic subsystem and the aviation logistics demand subsystem. Enhancements in regional economic conditions coincide with increases in foreign trade volume, total retail sales of consumer goods, and the resident population. These factors, both directly and indirectly, stimulate the growth in demand for aviation logistics. However, if the supply capacity is not adequately adjusted to meet the rising demand, a supply and demand imbalance arises, resulting in a decline in the supply and demand ratio. The unmet demand for aviation logistics, crucial for supporting regional industrial development and operations, can significantly impede industrial growth and severely restrict further regional economic development.

Loop 6: Aviation logistics demand level → -Aviation logistics supply and demand ratio → -Total funds for civil aviation development→+Development level of aviation logistics equipment hardware and software→+Aviation logistics supply capacity→+Aviation logistics service level→+Aviation logistics demand level
Loop 7: Aviation logistics supply capacity→+Aviation logistics supply and demand ratio → -Total funds for civil aviation development→+Development level of aviation logistics equipment hardware and software→+Aviation logistics supply capacity

Feedback loops 6 and 7 illustrate the interactions and affecting processes between the funds for civil aviation development and the balance of supply and demand in aviation logistics. The supply and demand ratio emphasizes the balance between the supply and demand levels in aviation logistics. Specifically, when the demand for aviation logistics increases without a corresponding rise in supply, the supply and demand ratio decreases. To enhance supply capacity and address the growing logistics demand driven by economic development, the government boosts investment in the aviation logistics sector. This, in turn, improves service quality, which stimulates higher demand for aviation logistics services. This increasing demand further influences the supply and demand ratio, generating endogenous momentum for supply expansion. Conversely, if supply exceeds demand and surpasses the balance point to a certain extent, it indicates an oversupply in aviation logistics. In such instances, the government may adjust its investment in the aviation logistics sector, reallocating resources to other critical areas.

### System flow diagram construction

The causal feedback model offers a qualitative depiction of the relationships among system components. To enhance the understanding of the logical interconnections and feedback mechanisms, this study expands the model by integrating level, rate, and auxiliary variables [42]. Following a thorough review of the existing literature [43,44], a total of 29 variables were selected, comprising 3 level variables, 3 rate variables, 13 auxiliary variables, and 10 constants and table functions. The primary variables are detailed in Table 2.

**Table 2:** Main variables of the simulation model.

Level variables	Rate variables	Primary auxiliary variables (Section)
Regional economic development level	Rate of increase in regional economic development level	Value added in the tertiary sector
		Aviation logistics transportation efficiency
		Aviation logistics supply and demand ratio
Aviation logistics demand level	Rate of increase in aviation logistics demand level	Total import and export value of foreign trade
		Total retail sales of consumer goods
		Regional resident population
		Aviation logistics service level
Aviation logistics supply capacity	Rate of increase in aviation logistics supply capacity	Development level of aviation logistics equipment hardware
		Development level of aviation logistics equipment software
		Total funds for civil aviation development

**Fig 4:**
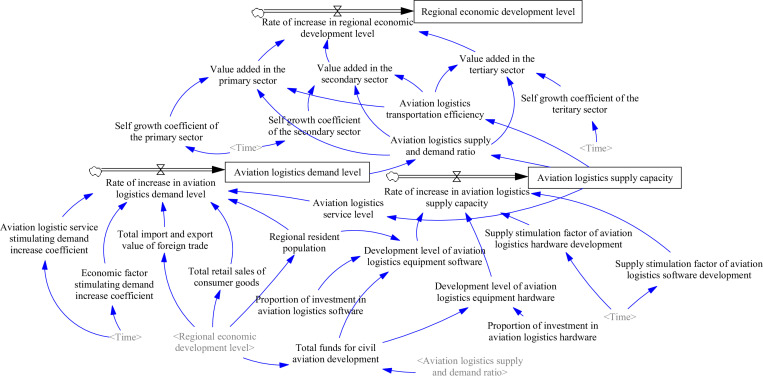
System flow diagram illustrating the mechanism through which aviation logistics drives regional economic development.

A system flow diagram was created using Vensim PLE 6.3 software, based on the interrelationships among the variables outlined above, as shown in Fig 4.

### Study area and data source

To validate the rationality and applicability of the model, this study selects Sichuan Province in China as the empirical region for several reasons:

(1) Sichuan Province’s regional economic development is significantly reliant on aviation logistics. Specifically, Sichuan is situated in the inland southwest of China, where its mountainous terrain and remote location pose considerable challenges for the timeliness of traditional transportation methods, such as road and rail. These geographical factors hinder transportation efficiency, complicating the province’s ability to meet its economic needs. As a result, Sichuan has increasingly depended on aviation logistics to address these challenges and promote economic growth. This strong dependence on aviation logistics renders the data from Sichuan particularly valuable for research, as it enhances the relevance and robustness of models investigating the role of aviation logistics in driving regional economic development.
(2) The Sichuan provincial government has prioritized the advancement of aviation logistics equipment, as detailed in the “14th Five-Year Plan for Modern Logistics Development in Sichuan Province” (2021). This plan underscores the significance of investing in aviation logistics equipment to improve operational efficiency. This study develops an SD model that considers the impact of aviation logistics equipment on regional economic development. This approach aligns closely with current policies in Sichuan that focus on the development of aviation logistics equipment, ensuring that the findings of this study will offer valuable insights for simulating the province’s current situation, forecasting future trends, and guiding policy adjustments.

Data for the SD model are primarily sourced from official statistics and authoritative databases, including the Statistical Yearbook of Sichuan Province (2013–2023), the Budget Expenditure and Final Accounts of the Government-Specific Funds at the Provincial Level of Sichuan (2013–2023), the Statistical Bulletin on the Development of the Civil Aviation Industry (2013–2023), and the CEIC Data database. The study utilizes real data from Sichuan Province for 2013–2023 and forecasts data for 2024–2033. This period was chosen based on data availability and relevant policy changes, as long-term planning in Sichuan and at the national level typically employs 10-year or longer periods (e.g., the 2035 vision outlined in the ‘14th Five-Year Plan’). The 2024–2033 forecast period aligns with national and regional planning cycles, supporting long-term regional economic objectives. The raw dataset and the projected data for Sichuan Province collected in this study can be found in S1 Table and S2 Table.

### Variables setting

This study integrates data estimation with the entropy method to determine variable weights for parameter setting and function creation.

Entropy is a fundamental concept that measures the degree of randomness or uncertainty associated with a given event [45]. The entropy method is widely acknowledged as an unbiased and precise approach for quantitative weighting [46]. The following outlines the specific steps involved in applying this method:

Step 1: Normalization method

Due to inconsistencies in the data and values collected for AL&RE indicators, it is essential to standardize these indicators to ensure their comparability. Therefore, the range normalization method is employed to convert the raw data of indicators with different scales into dimensionless values. Since all indicators in this study are positive, the positive normalization formula is utilized for this process:


$$R_{ij}=\frac{X_{ij}-X_{\min}}{X_{\max}-X_{\min}}$$ (1)

Here, ‘*i*’ represents the year, where in this study, *i* = 2013, 2014, …, 2033, and ‘*j*’ represents the indicators. ‘$X_{ij}$’ and ‘$R_{ij}$’ denote the original and standardized values of the *j*-th indicator in the *i*-th year, respectively. The ‘$X_{\max}$’ and ‘$X_{\min}$’ represent the maximum and minimum values of the *j*-th indicator in its original data.

Step 2: Contribution of each indicator

This step involves calculating the characteristic weight and contribution of each indicator that influences the development level of its target variable. The formula is as follows:


$$P_{ij}=\frac{X_{ij}}{\sum_{i=1}^{m}X_{ij}}$$ (2)

Step 3: Entropy value

In this step, the entropy value of each indicator to its target variable is calculated:


$$e_{j}=-\frac{1}{lnm}\sum_{i=1}^{m}P_{ij}\ln\left(P_{ij}\right),0\leq e_{j}\leq 1$$ (3)

Step 4: Weight of evaluation indicators

The formula is as follows:


$$W_{j}=\frac{1-e_{j}}{\sum_{j=1}^{n}\left(1-e_{j}\right)}$$ (4)

In univariate formulas, the ratio of the dependent to independent variables acts as a parameter. In multivariate cases, weights are assigned to the independent variables to identify the optimal parameters for the dependent variable formula. The sequence of the coefficient table is established using the same method. Parameter settings and model reliability are confirmed through repeated testing. The primary variable formulas are detailed in Table 3.

**Table 3:** The formulas of level, rate, and auxiliary variables.

Variable type	Variable name	Functional formula
Level variables	Regional economic development level	= INTEG (Rate of increase in regional economic development level, 2651.8)
	Aviation logistics demand level	= INTEG (Rate of increase in aviation logistics demand level, 4.1)
	Aviation logistics supply capacity	= INTEG (Rate of increase in aviation logistics supply capacity, 5.2)
Rate variables	Rate of increase in regional economic development level	= Value added in the primary sector+ Value added in the secondary sector+ Value added in the tertiary sector
	Rate of increase in aviation logistics demand level	= Regional resident population* Economic factor stimulating demand increase coefficient+ Total import and export value of foreign trade* Economic factor stimulating demand increase coefficient+ Total retail sales of consumer goods * Economic factor stimulating demand increase coefficient+ Aviation logistics service level* Aviation logistic service stimulating demand increase coefficient
	Rate of increase in aviation logistics supply capacity	= Supply stimulation factor of aviation logistics hardware development* Development level of aviation logistics equipment hardware+ Supply stimulation factor of aviation logistics software development* Development level of aviation logistics equipment software
Auxiliary variables	Value added in the primary sector	= Self growth coefficient of the primary sector* Aviation logistics transportation efficiency* IF THEN ELSE (Aviation logistics supply and demand ratio>= 1, 1, Aviation logistics supply and demand ratio)
	Value added in the secondary sector	= Self growth coefficient of the secondary sector* Aviation logistics transportation efficiency* IF THEN ELSE (Aviation logistics supply and demand ratio>= 1, 1, Aviation logistics supply and demand ratio)
	Value added in the tertiary sector	= Self growth coefficient of the tertiary sector* Aviation logistics transportation efficiency* Aviation logistics supply and demand ratio
	Aviation logistics supply and demand ratio	= Aviation logistics supply capacity/ Aviation logistics demand level
	Aviation logistics transportation efficiency	= Aviation logistics supply capacity* 0.011
	Aviation logistics service level	= Aviation logistics supply capacity* 0.002
	Total import and export value of foreign trade	= Regional economic development level* 0.0792
	Total retail sales of consumer goods	= Regional economic development level* 0.0626
	Regional resident population	= Regional economic development level* 0.0885
	Development level of aviation logistics equipment hardware	= Total funds for civil aviation development* Proportion of investment in aviation logistics hardware* 1.4286

This study references existing research [23,47] to assess regional economic development using GDP, evaluates aviation logistics supply through cargo throughput, and measures demand through aviation freight volumes.

This study assumes that the added value of industries changes naturally, without the influence of aviation logistics. Based on this natural change, the ability to meet their aviation transport demand and the efficiency of aviation transport can influence their added value. However, for the tertiary industry, aviation logistics is a crucial component that not only affects added value through transportation but also directly generates economic benefits from its activities.

Notably, the constants defined in this study will act as adjustment variables in subsequent sensitivity analysis, and a detailed interpretation is warranted, as detailed in Table 4.

**Table 4:** Formulas and interpretations of constants.

Variable type	Variable name	Functional formula	Interpretation
Constants	Proportion of investment in aviation logistics hardware	= 0.7	The proportion of investment in aviation logistics hardware was calculated based on the funding allocated for aviation logistics equipment hardware and the overall aviation logistics industry in Sichuan Province from 2013 to 2023. According to the calculation results, the initial value is set at 0.7, which can be adjusted to simulate various investment policies. Similarly, the proportion of investment in aviation logistics software was determined based on expenditures for equipment software and airport subsidies during the same period. The initial value for this proportion is set at 0.3, which can also be adjusted to reflect different investment strategies.
	Proportion of investment in aviation logistics software	= 0.3	

## Results and discussion

After developing the SD model, a simulation was conducted for the years 2013–2033, utilizing actual data from 2013 to 2023 and projected data from 2024 to 2033 for Sichuan Province. To gain a deeper understanding of the key factors and their effects on the regional economy, result and sensitivity analyses were performed.

### Model validation

Model validation is essential for ensuring the model’s reliability and accuracy. This process involves comparing the simulated values of selected variables with actual values to ensure that errors remain within acceptable limits [48]. The study utilizes regional economic development level and aviation logistics supply capacity as validation variables. Simulation data from 2013 to 2023 are compared with actual values, and the resulting errors are calculated, as shown in Tables 5 and 6.

**Table 5:** The regional economic development level validation results.

Year	Regional economic development level data comparison			
	Actual value	Stimulated value	Error ratio	Whether it conforms to
2013	2651.80	2651.80	0.00%	Yes
2014	2889.13	2889.13	0.00%	Yes
2015	3034.20	3034.20	0.00%	Yes
2016	3313.85	3313.85	0.00%	Yes
2017	3790.51	3790.51	0.00%	Yes
2018	4290.21	4290.21	0.00%	Yes
2019	4636.38	4636.37	0.00%	Yes
2020	4850.16	4850.14	0.00%	Yes
2021	5408.80	5408.78	0.00%	Yes
2022	5674.98	5661.02	-0.25%	Yes
2023	6013.29	6013.28	0.00%	Yes

**Table 6:** The aviation logistics supply capacity validation results.

Year	Aviation logistics supply capacity data comparison			
	Actual value	Stimulated value	Error ratio	Whether it conforms to
2013	5.17	5.17	0.00%	Yes
2014	5.62	5.62	0.00%	Yes
2015	5.74	5.74	0.00%	Yes
2016	6.32	6.32	0.00%	Yes
2017	6.62	6.62	0.00%	Yes
2018	6.80	6.80	0.00%	Yes
2019	6.99	6.99	0.00%	Yes
2020	6.46	6.46	0.00%	Yes
2021	6.80	6.80	0.00%	Yes
2022	6.30	6.30	0.00%	Yes
2023	7.98	7.98	0.00%	Yes

Tables 5 and 6 demonstrate that the error rates between the simulated and actual values of regional economic development and aviation logistics supply capacity are both below 10% [49]. This suggests that the model is accurate and dependable in simulating the AL&RE complex system and the influence of aviation logistics on regional economic development, effectively mirroring real conditions and forecasting future trends.

### Simulation results analysis

**Fig 5:**
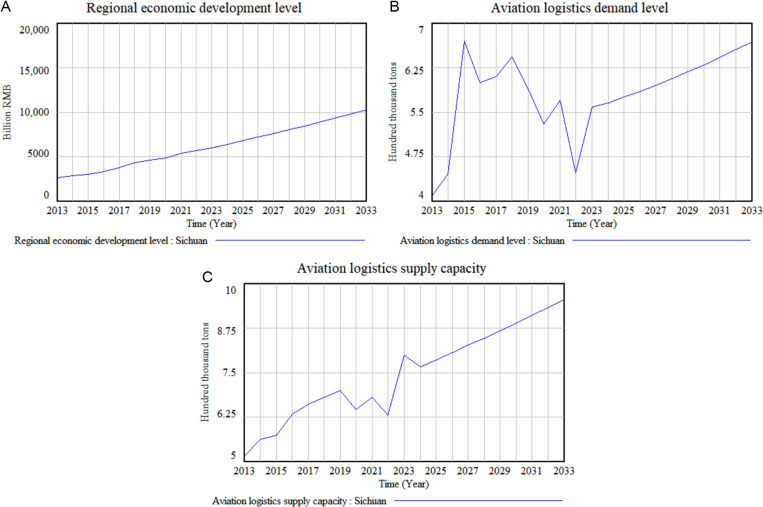
Trends in the development of the regional economy, aviation logistics demand, and aviation logistics supply in Sichuan Province.

To investigate the current development trends and future evolution of the AL&RE subsystems, this study concentrates on three level variables: ‘Regional economic development level,’ ‘Aviation logistics demand level,’ and ‘Aviation logistics supply capacity.’ These variables act as the main indicators for observation. The model produces output values for each level variable throughout the simulation cycle, as shown in Fig 5.

As shown in Fig 5(a), Sichuan Province’s regional economy has exhibited consistent growth from 2013 to 2023, with projections indicating continued steady growth through 2033, suggesting an optimistic economic outlook.

Fig 5(b) illustrates a steady increase in aviation logistics demand from 2013 to 2015, driven by industrial growth and infrastructure improvements. After peaking in 2015, demand fluctuated and declined by 2022, likely due to global economic slowdowns and the effects of the COVID-19 pandemic [50]. A recovery began in 2023, fueled by trade rebounds and post-pandemic economic recovery, with steady growth anticipated through 2033. The demand fluctuations underscore the sector’s vulnerability to external factors. This vulnerability arises from the sector’s dependence on elements such as international trade, social consumption activities, and other socio-economic behaviors, all of which are subject to inherent uncertainties. These factors contribute to the sector’s instability, with policy responses often being delayed or ineffective. Consequently, shifting economic cycles and external health crises can result in abrupt and unpredictable changes in demand patterns.

Fig 5(c) illustrates a consistent increase in aviation logistics supply capacity from 2013 to 2019, followed by fluctuations and a decline by 2022, largely due to the pandemic’s impact. However, it is noteworthy that these fluctuations are relatively milder compared to the more significant variations seen in aviation logistics demand. A recovery was noted in 2023, driven by the easing of the pandemic’s effects, the effectiveness of pandemic-related policies, and renewed investments in the context of economic recovery. A slight decrease is expected in 2024, likely due to policy adjustments or market recalibrations during the post-recovery phase, as well as in response to new development dynamics. Following stabilization through policy guidance, capacity is projected to grow steadily through 2033. This relative stability and moderate fluctuations in the face of external shocks reflect the adaptability of the aviation logistics supply subsystem, which may be primarily influenced by timely interventions from local governments. When signs of fluctuation arise, government policies can effectively guide recovery and development, enhancing the system’s resilience to external shocks.

The simulation results demonstrate that, in the dynamic system where aviation logistics drives regional economic development, although the supply and demand of aviation logistics exhibit varying degrees of fluctuation, the overall trend remains upward. This closely aligns with trends in regional economic development, thereby confirming the role of aviation logistics in fostering regional economic development. While the demand for aviation logistics shows significant susceptibility to external factors, the adaptability of supply capacity ensures that the overall operations and performance of aviation logistics remain in sync with regional economic needs. Overall, the positive trends in both demand and supply underscore aviation logistics as a key enabler of trade, consumer confidence, and investment, propelling economic growth across various sectors.

### Policy design and sensitivity analysis

Based on existing literature and simulation results, regional economic development is shaped by both aviation logistics demand and supply factors. Aviation logistics demand is limited by external elements such as market conditions and international influences, demonstrating significant unpredictability and uncontrollability [51]. In contrast, supply capacity is strongly affected by policy interventions. National and governmental initiatives to enhance aviation logistics supply capacity can be achieved through investments in both hardware components and software aspects. These investments stimulate aviation logistics advancement and encourage regional economic development.

Under resource constraints, a significant challenge is the efficient allocation of resources, emphasizing targeted investments in areas where aviation logistics can most effectively promote regional economic development. This study examines the critical factors that can drive high-quality and sustainable regional economic development. It primarily concentrates on the aviation logistics supply subsystem for policy simulation and analysis, taking into account the lower sensitivity and longer response time of aviation logistics demand to policy changes, in contrast to the greater susceptibility of supply.

The study formulates funding allocation policies for both the hardware and software components of the aviation logistics supply subsystem, grounded in the current regional policies and future development trends in Sichuan Province. It subsequently performs a sensitivity analysis to pinpoint the key factors that effectively promote high-quality and sustainable regional economic development.

### Policy scenario design

This study, assuming a fixed total fund for civil aviation development, simulates different funding allocation policies by varying the proportion of investment in aviation logistics equipment, both hardware and software. The aim is to investigate how diverse policies that emphasize hardware and software investments in aviation logistics equipment influence the development level and structure of the regional economy, thereby reflecting the differing impacts of these factors on high-quality and sustainable regional economic development. The specific policy simulation scenarios are detailed in Table 7.

**Table 7:** Policy simulation of the investment proportions in hardware and software components of aviation logistics equipment.

Policy scenario	Proportion of investment in aviation logistics equipment hardware	Proportion of investment in aviation logistics equipment software
Scenario 1	0.7	0.3
Scenario 2	0.5	0.5
Scenario 3	0.3	0.7

This study develops three funding allocation policies, as shown in Table 7, after thoroughly considering factors such as the current state of regional funding distribution and national and regional development trends. Specifically, the reasoning behind selecting these scenarios is as follows:

Scenario 1 (Proportion of investment in aviation logistics equipment hardware: 0.7, Proportion of investment in aviation logistics equipment software: 0.3): This funding allocation ratio is based on the actual distribution of funds in Sichuan Province during the study period, utilizing data from the ‘Budget Expenditure and Final Accounts of the Government-Specific Funds at the Provincial Level of Sichuan (2013-2023)’. It reflects the province’s historical focus on the development of logistics hardware in comparison to software systems. This scenario aims to replicate the current funding allocation status and serve as a baseline for future adjustments.

Scenario 2 (Proportion of investment in aviation logistics equipment hardware: 0.5, Proportion of investment in aviation logistics equipment software: 0.5): This scenario illustrates a balanced investment strategy in both hardware and software, reflecting the technological integration shift observed in recent years within the aviation logistics sector. As information technology and intelligent systems mature, the significance of software systems (such as talent development) has increased. A 50:50 funding split between hardware and software signifies a strategic shift towards fostering synergies between these two areas, ensuring that neither hardware infrastructure nor software capabilities remain underdeveloped. This balanced strategy aligns with broader industry trends toward technological convergence. It seeks to examine the potential impacts of such an investment distribution on regional economic development, especially as integrated technologies continue to transform the logistics landscape.

Scenario 3 (Proportion of investment in aviation logistics equipment hardware: 0.3, Proportion of investment in aviation logistics equipment software: 0.7): This scenario allocates a greater share of investment to software, reflecting the increasing focus on digital transformation and the integration of intelligent technologies within the aviation logistics sector. This strategy aligns with trends in digital transformation and strategic adjustments. Unlike Scenario 1, which emphasizes hardware development, this scenario underscores the vital role of software in propelling the future growth and evolution of the aviation logistics industry. It also aligns with China’s broader agenda of advancing digitalization and promoting the development of smart infrastructure.

The three scenarios illustrate distinct stages of investment prioritization in aviation logistics, reflecting both regional and national trends. Scenario 1 acts as a baseline, concentrating on physical logistics hardware. Scenario 2 promotes a balanced investment strategy, encouraging the concurrent development of software and hardware to integrate technology. Scenario 3 adopts a forward-looking perspective, highlighting digital transformation and software-driven innovation, in line with national objectives for smart logistics. Collectively, these scenarios offer a framework for understanding how different levels of investment in hardware and software can enhance the role of aviation logistics in fostering high-quality and sustainable regional economic development.

### Sensitivity analysis

This study evaluates the quality and sustainability of regional economic development based on economic output and industrial structure over a defined period. GDP reflects short-term economic performance, encompassing resource utilization, production efficiency, and productivity, with higher GDP indicating stronger economic activity and resilience. However, GDP alone is inadequate for assessing long-term development potential. Economic structure, which defines the composition of the regional economy, plays a vital role in future growth [52]. Regions with high-tech, innovation-driven industries tend to exhibit better long-term sustainability, while those reliant on traditional manufacturing may encounter limitations. The study analyzes changes in regional economic output and structural dynamics under various policy scenarios.

**Fig 6:**
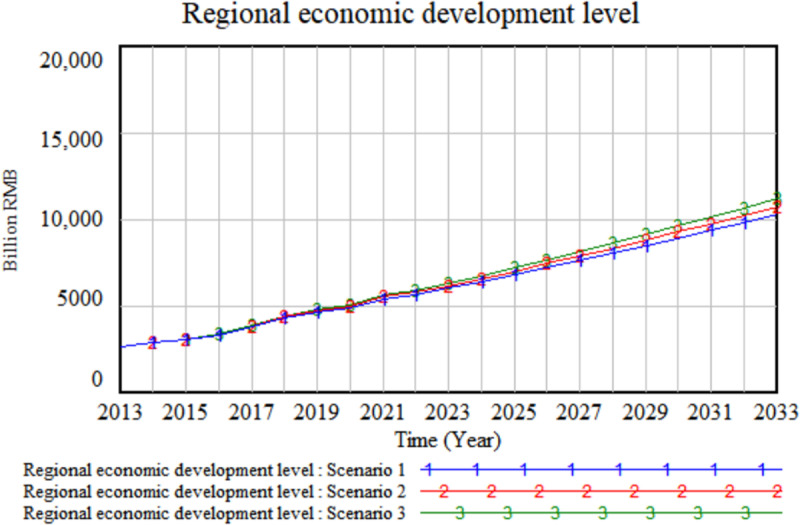
Simulation output on changes in regional economic development levels across three policy scenarios.

Based on the three policy scenarios, simulations were conducted on the level of regional economic development, which represents the quality of regional economic development. The results are presented in Fig 6.

Based on the simulation output of Fig 6, Scenario 3 emerges as the most optimal framework for regional economic development. This suggests that, compared to investments in hardware, the upgrading and optimization of software play a more significant role in driving regional economic development. Specifically, increasing investment in software for aviation logistics equipment proves to be more effective in enhancing regional economic development than focusing on hardware investments. This outcome can be attributed to the fact that, as digital transformation and intelligent systems continue to evolve, software optimization enhances operational efficiency, scalability, and adaptability, thereby creating greater long-term economic benefits. In contrast, while investments in hardware are foundational, they yield diminishing returns once they reach a certain level of maturity, limiting their impact on sustained economic value creation. Therefore, under the relatively mature framework of aviation logistics equipment hardware in Sichuan Province, which has long received strong support, prioritizing software development in aviation logistics equipment delivers more substantial and enduring contributions to economic advancement.

It is noteworthy that, over time, the growing significance of prioritizing the advancement of aviation logistics equipment software for regional economic development has become increasingly apparent compared to hardware investments. This temporal effect can be attributed to the ongoing digital transformation and the maturation of hardware equipment, which has resulted in diminishing returns from additional hardware investments. The digital trend is expected to persist, driving substantial changes in the aviation logistics industry. Therefore, adjusting policies to support the development of aviation logistics software can yield higher marginal benefits and further enhance regional economic development.

To summarize, Sichuan’s policy has long concentrated on developing aviation logistics equipment hardware, such as airports and fleet modernization, which has established a foundation for aviation logistics operations. However, as hardware has matured, the potential for further efficiency improvements has plateaued, resulting in a reduced impact on regional economic development. Therefore, adjusting policies to promote the development of aviation logistics software could yield greater marginal benefits and unlock more potential for high-quality regional economic development.

**Fig 7:**
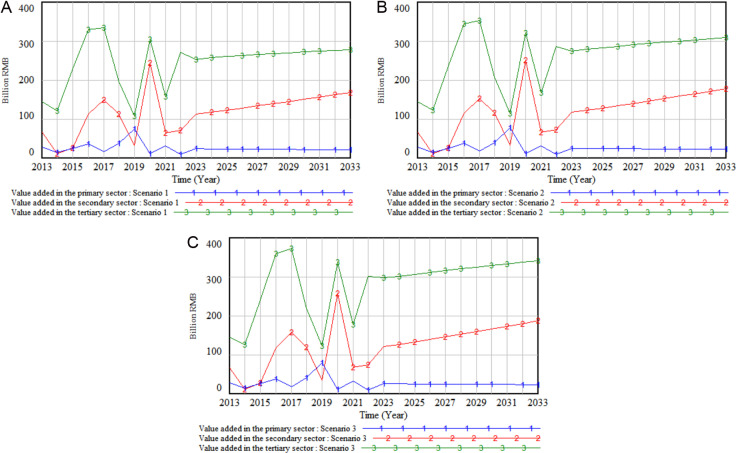
Simulation output on changes in regional economic structure across three policy scenarios.

Furthermore, to investigate the variations in regional economic sustainable development under different policy scenarios, a simulation was performed on the regional economic structure based on the three policy scenarios outlined in Table 7, with the results displayed in Fig 7.

As illustrated in Fig 7, heightened investment in aviation logistics equipment software has resulted in varying degrees of optimization in the industrial outputs of Sichuan Province, with value-added contributions increasing across all three sectors. The most notable improvement was seen in the tertiary sector, followed by the secondary sector, and then the primary sector. Importantly, under the policy framework of Scenario 3, the enhancement effect was most pronounced, with the largest increases in value-added noted across all three sectors. This indicates that prioritizing financial investment in aviation logistics equipment software has a significant positive impact on the development of all sectors, particularly the tertiary sector, which experienced the highest growth rate. This suggests that the increased investment in aviation logistics equipment software has optimized the regional economic structure, contributing to the overall advancement of the regional economic framework.

The observed developments can be attributed to enhancements in transportation efficiency and service quality, along with the inherent characteristics of the aviation logistics industry. Increased investment in aviation logistics software has bolstered the application of real-time technologies. Real-time analysis of factors such as demand, cargo location, and weather aids in reducing delays and optimizing flight utilization, thereby enhancing operational efficiency. Furthermore, real-time tracking ensures transparency and facilitates swift issue resolution, improving service quality and customer satisfaction. These advancements yield higher marginal benefits for industries, driving increased industrial output. Additionally, as aviation logistics is a vital component of the tertiary sector, its development directly contributes to the growth of this sector. The tertiary sector, particularly industries reliant on high-value, high-tech, and safety-sensitive products, is more dependent on aviation logistics compared to the primary and secondary sectors. Consequently, improvements in aviation logistics services have the most significant impact on increasing the value-added output within the tertiary sector.

### Policy recommendations

Based on the sensitivity analysis, Sichuan Province’s substantial investment in aviation logistics equipment hardware was initially advantageous, establishing a solid foundation. However, this ongoing investment has resulted in a trend toward saturation, reducing its marginal effect on high-quality and sustainable regional economic development. Consequently, it is advised that the province reevaluate its investment strategy, reallocating more resources toward software development to seize emerging opportunities. Specific measures include:

(1) Gradual reallocation of resources: Sichuan Province should prioritize the development of aviation logistics equipment software by gradually shifting a portion of the current hardware-focused investments toward software initiatives. This could involve fostering innovation in logistics management systems, data analytics, automation, and artificial intelligence to enhance operational efficiency. However, it is crucial to ensure that hardware investments are not abruptly reduced to prevent stagnation in the infrastructure. A gradual and balanced approach should be adopted to support both software and hardware development.
(2) Encourage public-private partnerships: To enhance the impact of software development, the province should foster collaborations among government agencies, software developers, and private enterprises. This could be achieved through joint research initiatives, pilot programs, and financial incentives, motivating the private sector to invest in the creation of innovative software solutions while the government offers regulatory support and funding.
(3) Incentivize technology startups and innovation hubs: Sichuan should establish a supportive environment for technology startups and innovation hubs centered on aviation logistics software. Providing tax incentives, grants, and research funding will stimulate entrepreneurial ventures in the software sector, nurturing a competitive and innovative ecosystem. Furthermore, forming partnerships with universities and research institutions can lead to groundbreaking advancements in software technologies.
(4) Continuous monitoring and evaluation: The government should implement a system for ongoing monitoring and evaluation of the aviation logistics sector’s development, particularly emphasizing the impact of software investments. Regular assessments of both hardware and software components should be conducted to gauge their effectiveness, identify bottlenecks, and ensure optimal resource utilization. Annual reports should be generated to inform future adjustments.

## Conclusions

This study develops a complex system model for AL&RE to analyze the interactions among subsystems and their internal components. A significant contribution of this research is the integration of aviation logistics equipment—an area of considerable national policy focus—into the model, exploring its role and positioning within the broader system. By defining and classifying aviation logistics equipment from a complex system perspective, the study fills a gap in the existing literature. To further investigate the dynamic mechanisms and key factors through which aviation logistics drives regional economic development, an SD model is constructed, utilizing empirical data from Sichuan Province to assess its applicability. Finally, the study simulates policy scenarios related to aviation logistics supply, conducting sensitivity analysis to identify critical factors that influence high-quality and sustainable regional economic development through aviation logistics. The key findings are as follows:

(1) The trends in the aviation logistics supply and demand subsystems, along with the regional economic subsystem, show a general consistency throughout the simulation period. This finding supports the potential of aviation logistics to drive regional economic development.
(2) Aviation logistics equipment is a vital element of aviation logistics supply and is greatly affected by policy regulations. These regulations enhance its relative stability and are instrumental in promoting regional economic development.
(3) Software components of aviation logistics equipment are more effective in promoting regional economic development than hardware, and they also play a more significant role in optimizing the regional economic structure. Overall, supporting aviation logistics software is more advantageous for high-quality and sustainable regional economic development.

Despite these findings, the study has some limitations. First, the research on the role of aviation logistics in driving regional economic development relies on a univariate analysis. However, changes in these variables may result in fluctuations in the level of coordinated development between AL&RE. Future studies could incorporate a coupling coordination model to further examine how such coordination levels change under sensitivity analysis, while also considering the influence of key factors. Additionally, although the study identifies that investment in aviation logistics software has a stronger driving effect on the regional economy compared to hardware, it does not establish the optimal investment ratio. Future research could develop more detailed input-output models to explore the ideal ratio of software and hardware investment at different stages of regional economic development, aiming to maximize the driving effect of aviation logistics on the regional economy.

## Supporting information

S1 Table Raw data of the regional economy subsystem in Sichuan Province. (DOCX)

S2 Table Raw data of the aviation logistics subsystem in Sichuan Province. (DOCX)

## References

[1] Sun Z. A study on the evaluation of competitiveness in the aviation logistics industry cluster in Zhengzhou. Sci Rep. 2024;14(1):2659. doi: 10.1038/s41598-024-52697-x 38302624 PMC10834970

[2] Zhao B, Wu H. A System Dynamics Model of Multi-Airport Logistics System under the Impact of COVID-19: A Case of Jing-Jin-Ji Multi-Airport System in China. Sustainability. 2022;14(19):12823. doi: 10.3390/su141912823

[3] Çelik AK, Yalçınkaya Ö, Kutlu M. The causal relationship between air transport and economic growth: Evidence from top ten countries with the largest air transport volume. Transport Policy. 2025;162:521–32. doi: 10.1016/j.tranpol.2025.01.002

[4] Delgado F, Sirhan C, Katscher M, Larrain H. Recovering from demand disruptions on an air cargo network. Journal of Air Transport Management. 2020;85:101799. doi: 10.1016/j.jairtraman.2020.101799

[5] WANG Y, Liu D, Sui X, Li F. Does logistics efficiency matter? Evidence from green economic efficiency side. Research in International Business and Finance. 2022;61:101650. doi: 10.1016/j.ribaf.2022.101650

[6] Ji X, Zhai Y, Fu S, Lu C. Towards the sustainable development of logistics system model: A system dynamics approach. PLoS One. 2023;18(1):e0279687. doi: 10.1371/journal.pone.0279687 36701292 PMC9879409

[7] Zhang F, Graham DJ. Air transport and economic growth: a review of the impact mechanism and causal relationships. Transport Reviews. 2020;40(4):506–28. doi: 10.1080/01441647.2020.1738587

[8] Sheard N. Airport Size and Urban Growth. Economica. 2018;86(342):300–35. doi: 10.1111/ecca.12262

[9] Chen X, Xuan C, Qiu R. Understanding spatial spillover effects of airports on economic development: New evidence from China’s hub airports. Transportation Research Part A: Policy and Practice. 2021;143:48–60. doi: 10.1016/j.tra.2020.11.013

[10] Zhou J, Leng L, Shi X. The Impact of Air Cargo on Regional Economic Development: Evidence from Chinese Cities. Sustainability. 2022;14(16):10336. doi: 10.3390/su141610336

[11] Njoya ET, Ragab AM. Economic Impacts of Public Air Transport Investment: A Case Study of Egypt. Sustainability. 2022;14(5):2651. doi: 10.3390/su14052651

[12] He H, Wu H, Tsui KWH, Wang B, Fu X. Spatiotemporal evolution of air cargo networks and its impact on economic development - An analysis of China’s domestic market before and during the COVID-19 pandemic. Journal of Transport Geography. 2024;117:103872. doi: 10.1016/j.jtrangeo.2024.103872

[13] Li H, Li J, Zhao X, Kuang X. The morphological structure and influence factors analysis of China’s domestic civil aviation freight transport network. Transport Policy. 2022;125:207–17. doi: 10.1016/j.tranpol.2022.06.008

[14] Hakim MM, Merkert R. Econometric evidence on the determinants of air transport in South Asian countries. Transport Policy. 2019;83:120–6. doi: 10.1016/j.tranpol.2017.12.003

[15] Torres L, Blevins AS, Bassett D, Eliassi-Rad T. The Why, How, and When of Representations for Complex Systems. SIAM Rev. 2021;63(3):435–85. doi: 10.1137/20m1355896

[16] Guzzo D, Pigosso DCA, Videira N, Mascarenhas J. A system dynamics-based framework for examining Circular Economy transitions. Journal of Cleaner Production. 2022;333:129933. doi: 10.1016/j.jclepro.2021.129933

[17] Rocha LEC. Dynamics of air transport networks: A review from a complex systems perspective. Chinese Journal of Aeronautics. 2017;30(2):469–78. doi: 10.1016/j.cja.2016.12.029

[18] He C, Wang C. Development model simulation of airport transport corridor based on system dynamics algorithm. Cluster Comput. 2018;22(S4):8225–39. doi: 10.1007/s10586-018-1728-8

[19] Peng Q, Wan L, Zhang T, Wang Z, Tian Y. A System Dynamics Prediction Model of Airport Environmental Carrying Capacity: Airport Development Mode Planning and Case Study. Aerospace. 2021;8(12):397. doi: 10.3390/aerospace8120397

[20] Anderson P W. The economy as an evolving complex system. CRC Press. 2018.

[21] Gao C, Gao C, Song K, Fang K. Pathways towards regional circular economy evaluated using material flow analysis and system dynamics. Resources, Conservation and Recycling. 2020;154:104527. doi: 10.1016/j.resconrec.2019.104527

[22] Jiang L, Zuo Q, Ma J, Zhang Z. Evaluation and prediction of the level of high-quality development: A case study of the Yellow River Basin, China. Ecological Indicators. 2021;129:107994. doi: 10.1016/j.ecolind.2021.107994

[23] Saidi S, Mani V, Mefteh H, Shahbaz M, Akhtar P. Dynamic linkages between transport, logistics, foreign direct Investment, and economic growth: Empirical evidence from developing countries. Transportation Research Part A: Policy and Practice. 2020;141:277–93. doi: 10.1016/j.tra.2020.09.020

[24] Amin C, Wahab Hasyim A, Sun’an M, , Millanida Hilman R, Fahmiasari H. Impact of increasing local economic capacity on reducing maritime logistics costs in island Province of eastern Indonesia: A dynamic system approach. Transportation Research Interdisciplinary Perspectives. 2024;27:101195. doi: 10.1016/j.trip.2024.101195

[25] Muecklich N, Sikora I, Paraskevas A, Padhra A. Safety and reliability in aviation – A systematic scoping review of normal accident theory, high-reliability theory, and resilience engineering in aviation. Safety Science. 2023;162:106097. doi: 10.1016/j.ssci.2023.106097

[26] Talwar C, Joormann I, Ginster R, Spengler T S. A system dynamics model of the air transport system. 2021. doi: 10.24355/DBBS.084-202105100741-0

[27] Chen L, Li X, Zhao J, Kang X, Liu L, Wang M, et al. Coupling and coordinated evolution characteristics of regional economy-energy-carbon emission multiple systems: A case study of main China’s Basin. J Environ Sci (China). 2024;140:204–18. doi: 10.1016/j.jes.2023.07.007 38331501

[28] Park S-Y, Wang X, Oh Y, Hong S-M, Woo S-H. Application of structural topic modeling in a literature review of air transport. Journal of Air Transport Management. 2025;122:102708. doi: 10.1016/j.jairtraman.2024.102708

[29] Gu Y, Wu Y, Xu M, Mu X, Zuo T. Waste electrical and electronic equipment (WEEE) recycling for a sustainable resource supply in the electronics industry in China. Journal of Cleaner Production. 2016;127:331–8. doi: 10.1016/j.jclepro.2016.04.041

[30] Govindan K, Mina H, Alavi B. A decision support system for demand management in healthcare supply chains considering the epidemic outbreaks: A case study of coronavirus disease 2019 (COVID-19). Transp Res E Logist Transp Rev. 2020;138:101967. doi: 10.1016/j.tre.2020.101967 32382249 PMC7203053

[31] Wu P, Yang C. Sustainable development in aviation logistics: Successful drivers and business strategies. Bus Strat Env. 2021;30(8):3763–71. doi: 10.1002/bse.2838

[32] Karpun O, Yakovenko V. The latest approaches and technologies to increase the competitiveness of aviation enterprises in modern conditions. Intellectualization of logistics and SCM. 2024;(23):44–53. doi: 10.46783/smart-scm/2024-23-4

[33] Tian J, Shen C, Wang B, Ren C, Xia X, Dong R, et al. EVADE: Targeted Adversarial False Data Injection Attacks for State Estimation in Smart Grid. IEEE Trans Sustain Comput. 2024:1–13. doi: 10.1109/tsusc.2024.3492290

[34] Tian J, Shen C, Wang B, Xia X, Zhang M, Lin C, et al. LESSON: Multi-Label Adversarial False Data Injection Attack for Deep Learning Locational Detection. IEEE Trans Dependable and Secure Comput. 2024;21(5):4418–32. doi: 10.1109/tdsc.2024.3353302

[35] Larrodé E, Muerza V, Villagrasa V. Analysis model to quantify potential factors in the growth of air cargo logistics in airports. Transportation Research Procedia. 2018;33:339–46. doi: 10.1016/j.trpro.2018.10.111

[36] Song M, Lin Y. Research on training strategy of logistics professional undergraduate talents. World Education Forum. 2024; 2(5). 10.70711/wef.v2i5.5059

[37] Rebs T, Brandenburg M, Seuring S. System dynamics modeling for sustainable supply chain management: A literature review and systems thinking approach. Journal of Cleaner Production. 2019;208:1265–80. doi: 10.1016/j.jclepro.2018.10.100

[38] Malbon E, Parkhurst J. System dynamics modelling and the use of evidence to inform policymaking. Policy Studies. 2022;44(4):454–72. doi: 10.1080/01442872.2022.2080814

[39] Al-Aziz FN, Suryani E. System Dynamics Modeling to Increase the Productivity of Chili Pepper through Good Agricultural Practices in East Java. Procedia Computer Science. 2024;234:733–40. doi: 10.1016/j.procs.2024.03.094

[40] Ibarra Vega D, Bautista-Rodriguez S. The impact of circular economy strategies on municipal waste management: A system dynamics approach. Cleaner Engineering and Technology. 2024;21:100761. doi: 10.1016/j.clet.2024.100761

[41] Zhou X, Hou J, Song Q, Wang Y. Exploring the Relationship Between Population Changes and Logistics Development: An Analysis Based on the Spatiotemporal Evolution Characteristics of Population and Logistics Coupling Coordination. Sustainability. 2024;17(1):93. doi: 10.3390/su17010093

[42] Davies K, Hart E, Galloway S. Quantifying impacts of sustainable transport interventions in Scotland: A system dynamics approach. Transportation Research Part D: Transport and Environment. 2024;133:104311. doi: 10.1016/j.trd.2024.104311

[43] Li X, Chen F. Impact of Logistics Development on Economic Growth: An Empirical Research from Guangdong Province in China. Complexity. 2021;2021(1). doi: 10.1155/2021/9950935

[44] Lili M. Research on the Relationship between Regional Logistics Demand and Service Innovation based on Optimal Allocation of Factors in Digital Economy. 2021 IEEE 2nd International Conference on Big Data, Artificial Intelligence and Internet of Things Engineering (ICBAIE). 2021:825–8. doi: 10.1109/icbaie52039.2021.9389872

[45] Gorgij AD, Moayeri MM. Proposing a novel method for the irrigation water quality assessment, using entropy weighted method, entitled: “EIWQI”. Environ Earth Sci. 2023;82(20). doi: 10.1007/s12665-023-11150-4

[46] Chen C-H. A Novel Multi-Criteria Decision-Making Model for Building Material Supplier Selection Based on Entropy-AHP Weighted TOPSIS. Entropy (Basel). 2020;22(2):259. doi: 10.3390/e22020259 33286032 PMC7516705

[47] Choi JH. A study on the change in the significance of GDP as a determinant of air demand - Discussions on brand-new air transport items. Transport Policy. 2023;133:186–97. doi: 10.1016/j.tranpol.2023.01.004

[48] Coletta VR, Pagano A, Zimmermann N, Davies M, Butler A, Fratino U, et al. Socio-hydrological modelling using participatory System Dynamics modelling for enhancing urban flood resilience through Blue-Green Infrastructure. J Hydrol (Amst). 2024;636:131248. doi: 10.1016/j.jhydrol.2024.131248 39416471 PMC7616713

[49] Liu J, Liu Y, Wang X. An environmental assessment model of construction and demolition waste based on system dynamics: a case study in Guangzhou. Environ Sci Pollut Res Int. 2020;27(30):37237–59. doi: 10.1007/s11356-019-07107-5 31893359

[50] Dey Tirtha S, Bhowmik T, Eluru N. An airport level framework for examining the impact of COVID-19 on airline demand. Transp Res Part A Policy Pract. 2022;159:169–81. doi: 10.1016/j.tra.2022.03.014 35313726 PMC8926924

[51] Bolić T, Castelli L, Corolli L, Scaini G. Flexibility in strategic flight planning. Transportation Research Part E: Logistics and Transportation Review. 2021;154:102450. doi: 10.1016/j.tre.2021.102450

[52] Constantine C. Economic structures, institutions and economic performance. Economic Structures. 2017;6(1). doi: 10.1186/s40008-017-0063-1

