# Supplementary material for: A simulation-based empirical study on the role of aviation logistics in driving high-quality and sustainable regional economic development: Focusing on dynamic mechanisms and key factors
Source: PLoS One. 2025 May 8;20(5):e0323110. doi: 10.1371/journal.pone.0323110 (PMC12061396; doi:10.1371/journal.pone.0323110)
Supplement: S1 Table — (DOCX) [file pone.0323110.s001.docx]

**S1 Table. Raw data of the regional economy subsystem in Sichuan Province.**

| **Year** | **GDP (Billion RMB)** | **Value added of the primary industry (Billion RMB)** | **Value added of the secondary industry (Billion RMB)** | **Value added of the tertiary industry (Billion RMB)** | **Total retail sales of consumer goods (Billion RMB)** | **Total import and export volume (Billion RMB)** | **Resident population (10,000 persons)** |
| --- | --- | --- | --- | --- | --- | --- | --- |
| 2013 | 26518.02 | 3257.42 | 12418.94 | 10841.7 | 10976.63 | 4008.06 | 8109 |
| 2014 | 28891.33 | 3524.74 | 13082.69 | 12283.9 | 12353.94 | 4314.75 | 8139 |
| 2015 | 30342.01 | 3660.96 | 13192.45 | 13488.6 | 13834.40 | 3172.80 | 8196 |
| 2016 | 33138.48 | 3900.60 | 13450.13 | 15787.8 | 15519.69 | 3261.45 | 8251 |
| 2017 | 37905.14 | 4262.51 | 14569.17 | 19073.5 | 17404.45 | 4604.87 | 8289 |
| 2018 | 42902.10 | 4427.43 | 16056.94 | 22417.7 | 19340.70 | 5946.71 | 8321 |
| 2019 | 46363.75 | 4807.50 | 17187.90 | 24368.3 | 21343.00 | 6789.83 | 8351 |
| 2020 | 48501.64 | 5556.58 | 17505.60 | 25439.2 | 20824.87 | 8088.64 | 8371 |
| 2021 | 54088.00 | 5661.86 | 19949.70 | 28476.2 | 24133.21 | 9395.60 | 8372 |
| 2022 | 56610.20 | 5965.50 | 20591.40 | 30053.3 | 24104.64 | 10044.30 | 8374 |
| 2023 | 60132.90 | 6056.60 | 21306.70 | 32769.5 | 26313.40 | 9574.91 | 8368 |
| 2024 | 63547.02 | 6477.53 | 22054.24 | 35015.1 | 27966.19 | 10723.46 | 8453 |
| 2025 | 67086.77 | 6783.16 | 23013.11 | 37290.3 | 29503.76 | 11462.19 | 8481 |
| 2026 | 70626.53 | 7088.78 | 23971.97 | 39565.6 | 31041.32 | 12200.92 | 8508 |
| 2027 | 74166.28 | 7394.40 | 24930.83 | 41840.8 | 32578.88 | 12939.64 | 8536 |
| 2028 | 77706.03 | 7700.03 | 25889.70 | 44116.1 | 34116.45 | 13678.37 | 8564 |
| 2029 | 81245.78 | 8005.65 | 26848.56 | 46391.3 | 35654.01 | 14417.10 | 8592 |
| 2030 | 84785.54 | 8311.27 | 27807.43 | 48666.6 | 37191.57 | 15155.83 | 8620 |
| 2031 | 88325.29 | 8616.90 | 28766.29 | 50941.8 | 38729.14 | 15894.56 | 8648 |
| 2032 | 91865.04 | 8922.52 | 29725.15 | 53217.1 | 40266.70 | 16633.29 | 8676 |
| 2033 | 95404.79 | 9228.14 | 30684.02 | 55492.3 | 41804.27 | 17372.02 | 8703 |
