# Supplementary material for: A simulation-based empirical study on the role of aviation logistics in driving high-quality and sustainable regional economic development: Focusing on dynamic mechanisms and key factors
Source: PLoS One. 2025 May 8;20(5):e0323110. doi: 10.1371/journal.pone.0323110 (PMC12061396; doi:10.1371/journal.pone.0323110)
Supplement: S2 Table — (DOCX) [file pone.0323110.s002.docx]

**S2 Table. Raw data of the aviation logistics subsystem in Sichuan Province.**

| **Year** | **Cargo turnover (Billion ton-kilometers)** | **Cargo throughput (100,000 tons)** | **Cargo transport volume (100,000 tons)** | **Employment in aviation logistics (10,000 persons)** | **Government aviation development fund expenditure (Billion RMB)** |
| --- | --- | --- | --- | --- | --- |
| 2013 | 8.0 | 5.17 | 4.10 | 3.5328 | 4.6850 |
| 2014 | 11.0 | 5.62 | 4.46 | 4.1694 | 5.1992 |
| 2015 | 11.7 | 5.74 | 6.70 | 4.2815 | 6.2651 |
| 2016 | 13.0 | 6.32 | 6.00 | 3.6664 | 7.7863 |
| 2017 | 14.0 | 6.62 | 6.10 | 3.8394 | 28.4478 |
| 2018 | 14.2 | 6.80 | 6.43 | 5.1954 | 58.2649 |
| 2019 | 12.8 | 6.99 | 5.90 | 3.5845 | 22.6288 |
| 2020 | 12.8 | 6.46 | 5.30 | 3.7440 | 11.8449 |
| 2021 | 14.5 | 6.80 | 5.70 | 4.0536 | 9.3020 |
| 2022 | 13.0 | 6.30 | 4.47 | 4.1834 | 10.2018 |
| 2023 | 14.3 | 7.98 | 5.59 | 4.3487 | 7.5049 |
| 2024 | 15.2 | 7.56 | 5.68 | 4.2373 | 18.1311 |
| 2025 | 15.6 | 7.75 | 5.71 | 4.2678 | 18.5449 |
| 2026 | 16.0 | 7.94 | 5.73 | 4.2983 | 18.9587 |
| 2027 | 16.5 | 8.12 | 5.76 | 4.3287 | 19.3725 |
| 2028 | 16.9 | 8.31 | 5.79 | 4.3592 | 19.7863 |
| 2029 | 17.3 | 8.50 | 5.81 | 4.3897 | 20.2001 |
| 2030 | 17.7 | 8.69 | 5.84 | 4.4201 | 20.6139 |
| 2031 | 18.1 | 8.87 | 5.87 | 4.4506 | 21.0277 |
| 2032 | 18.6 | 9.06 | 5.89 | 4.4811 | 21.4415 |
| 2033 | 19.0 | 92.5 | 59.2 | 4.5116 | 21.8554 |
